# Assessment of the performance of early warning scores in patients with COVID-19

**DOI:** 10.1590/1980-220X-REEUSP-2024-0371en

**Published:** 2025-05-26

**Authors:** Victoria Castilho Bartolomeu, Luiz Humberto Vieri Piacezzi, Karina Aparecida Lopes da Costa, Ruth Ester Assayag Batista, Cássia Regina Vancini Campanharo, Maria Carolina Barbosa Teixeira Lopes

**Affiliations:** 1 Universidade Federal de São Paulo, Escola Paulista de Enfermagem, São Paulo, SP, Brazil.; 2 Universidade Federal de São Paulo, Escola Paulista de Enfermagem, Departamento de Enfermagem Clínica e Cirúrgica, São Paulo, SP, Brazil.

**Keywords:** Triage, Early Warning Score, COVID-19, Emergency Medical Services, Nursing Care

## Abstract

**Objective:**

To assess the performance of the early warning scores Modified Early Warning Score, National Early Warning Score 2, National Early Warning Score Age and quick Sepsis Related Organ Failure Assessment for identifying clinical deteriorations in patients with COVID-19.

**Method:**

A retrospective cohort study. Patients with confirmed diagnosis of COVID-19 were included. Clinical deteriorations were acute respiratory failure, shock and cardiorespiratory arrest, and the outcomes were unscheduled admission to the Intensive Care Unit, discharge and death. The sensitivity and probability of a positive result in patients (true positive) and the specificity and probability of a negative result in non-patients (true negative) were determined.

**Results:**

The National Early Warning Score 2 and the National Early Warning Score Age showed better sensitivity values for early identification of deteriorations and outcomes.

**Conclusion:**

NEWS 2 and NEWS A showed better sensitivity and specificity, and their use is recommended to support the clinical assessment of patients with COVID-19. This study contributes to healthcare practice by assessing scores that can improve clinical assessment, allowing rapid identification of signs of deterioration in patients affected by COVID-19 and early interventions.

## INTRODUCTIOn

Emergency services (ES) play a fundamental role in access to the Brazilian Healthcare system and are one of the main means of access to healthcare for the Brazilian population^(1)^. Worldwide, there is an increase in demand for ES, so this is not a new phenomenon and its causes are multifactorial, such as increase in life expectancy and incidence of chronic non- communicable diseases, poverty and urban violence, in addition to the possibility of rapid access to diagnostic medicine and complex treatments in a single place^(2)^.

In the context of the ES, nurses act as a leading figure for clinical observation and early detection of clinical worsening, especially in the pandemic scenario, in which the clinical condition could deteriorate rapidly. Thus, with the support of the early warning score (EWS), it is possible to improve health safety and, consequently, hospital indicators^(2)^.

The COVID-19 pandemic has increased pressure on ES, which now receive approximately 10.3% more users compared to previous years^(3)^. In response, health centers expanded their supply of beds, material and human resources, encouraging or implementing flows that increased the surveillance and safety of admitted patients^(4)^, such as EWS, which began to be used in this context^(5)^.

EWS are models that assign scores according to physiological variables measured at the bedside, stratifying the risk of clinical deterioration, which makes it possible to trigger early healthcare^(6)^. Over time, modifications and adaptations made to these models gave rise to different types of EWS, validated in different scenarios^(7)^. Studies show that the use of early identification scores for clinical deterioration support clinical assessment in a more assertive manner, providing standardized communication about health status and reducing mortality^(8)^.

Given the various scores developed and adapted, it is essential to identify the most accurate EWS for use in the practice of screening clinical deterioration, since COVID-19 often causes “silent hypoxemia”, an event in which there is little change in other physiological parameters^(9)^. Therefore, this study aimed to assess the performance of Modified Early Warning Score (MEWS), quick Sequential Organ Failure Assessment (qSOFA), National Early Warning Score version 2 (NEWS 2) and National Early Warning Score Age (NEWS A) in relation to the ability to identify early clinical deterioration (acute respiratory failure, shock and cardiopulmonary arrest), admission to the Intensive Care Unit (ICU), and outcome discharge and death in patients admitted to the ES with a diagnosis of COVID-19. This study contributes to the implementation of evidence-based nursing care, as it demonstrates how scores are support tools for nurses’ clinical decision-making in the monitoring of hospitalized patients, especially in dynamic environments such as ES, contributing to the reduction of health problems and mortality.

## METHOD

### Study Design, Period and Location

This is a retrospective cohort study whose methodological steps were guided by the STrengthening the Reporting of OBservational studies in Epidemiology (STROBE) tool, carried out in a large university hospital^(10)^ between July 2022 and May 2023. The ES, during the COVID-19 pandemic, established a specific risk classification room for patients with respiratory symptoms and a respiratory failure unit for suspected or confirmed cases of COVID-19 who required hospitalization.

### Population

Secondary data from patients over 18 years of age admitted to the ES with a confirmed diagnosis of COVID-19 through Reverse Transcription Polymerase Chain Reaction testing between April and August 2020 and who agreed to participate in the study were used. Patients with incomplete data for calculating scores and pregnant women were excluded.

### Data Collection

The data were obtained through analysis of electronic medical records and comprised sociodemographic variables (age, sex, skin color, and education), comorbidities, signs and symptoms, risk classification category, vital signs for calculating scores at the time of admission, occurrence of clinical deterioration, severity markers, and outcomes (discharge and death).

In this study, the following were considered clinical deterioration: (1) acute respiratory failure (ARF), characterized by the introduction or increase in oxygen titration or escalation in the ventilatory mode; (2) shock, defined by the introduction of a new vasoactive drug; and (3) cardiorespiratory arrest (CRA). The EWS assessed were MEWS, qSOFA, NEWS 2 and NEWS A.

The MEWS scores from 1 to 3 (with 1 being the expected score for patterns within normality, and 3 being the score for altered patterns) the following parameters: heart rate (HR), with 0: 51–100 bpm, 1: 101–110 bpm, 2: 111–130 bpm and 3: > 130 bpm or < 40 bpm; respiratory rate (RR) being 0: 12–20 ipm, 1: 21–24 ipm, 2: 25–29 ipm and 3: > 30 ipm; temperature (T), being 0: 36.1 ºC–38 ºC, 1: < 36 ºC or > 38 ºC and 2: ≥ 39.1 ºC; systolic blood pressure (SBP), being 0: > 100 mmHg, 1: 81–100 mmHg, 2: 71–80 mmHg and 3: < 70 mmHg; and level of consciousness, according to the Glasgow Coma Scale (GCS), being 0: GCS 15, 1: GCS 14-13, 2: GCS 12–9 and 3: GCS < 9. The sum of the scores ranges from 0 to 14, and we characterize high clinical risk ≥ 4 points^(11)^, since this result tends to point to a risk of shock, respiratory failure, cardiovascular failure, metabolic acidosis, hypoxia and neurological deterioration^(11)^, requiring more rigorous monitoring and team intervention.

qSOFA, a widely used score for sepsis screening, assesses RR, with 1 point for RR > 22 bpm, SBP, with 1 point for SBP ≤ 100 mmHg, and change in level of consciousness, with 1 point for disorientation or decreased response to stimuli. The sum of the scores ranges from 0 to 3, and a low risk for sepsis is considered to be 0-1 point and a high risk of 2-3 points^(12)^, requiring urgent assessment and immediate interventions.

NEWS 2 can range from 0 to 20 points, with a higher score indicating a higher risk of clinical deterioration, and is composed of the following parameters: RR (12–≥ 30 ipm); HR (51–> 130 bpm); oxygen saturation (≥ 96%–≤ 91%); need for additional oxygen support; SBP (≥ 100 mmHg–≤ 80 mmHg); and level of consciousness (GCS 15–< 9)^(13)^. NEWS A uses the same parameters as NEWS 2, adding 3 points for patients aged 65 or over^(14)^. We categorized clinical risk as low risk (0–4), moderate risk (5–6) and high risk (≥ 7), as recommended by the Royal College of Physicians^(15)^. Individuals with low risk, but who received 3 points in some category, were considered moderate risk^(15)^.

### Analysis of Results, and Statistics

The data were stored on the Research Electronic Data Capture (REDCap)^®^ platform. For continuous variables, the mean, standard deviation, median, minimum and maximum were calculated, and for categorical variables, frequency and percentage were calculated. The performance of scores was calculated using sensitivity, which is the probability of a positive result in patients (true positive), and specificity, which is the probability of a negative result in non-patients (true negative).

Complementary analysis was performed using the Kappa coefficient of agreement, interpreted according to Altman^(16)^ as poor (< 0.2), reasonable (0.21 to 0.4), moderate (0.41 to 0.6), good (0.61 to 0.8) and very good (0.8 to 1). Fleiss’s Kappa was calculated to analyze the agreement between the four scores and clinical deterioration, and Cohen’s Kappa was calculated between the scores individually.

### Ethical Aspects

The study was guided by Resolution 466/2012 and approved by the *Universidade Federal de São Paulo* Research Ethics Committee, under Opinion 4,087,059. The Informed Consent Form was obtained online.

## RESULTS

A total of 356 patients were included, the majority of whom were men, white, with incomplete elementary education and with comorbidities (Table 1). Hypertension was the most frequent comorbidity (n = 211, 59.3%), followed by diabetes mellitus (n = 137, 38.5%), heart disease (n = 76, 21.3%) and solid organ transplant (n = 70, 19.7%).

**Table 1:** Sociodemographic variables and presence of comorbidities in the study population – São Paulo, SP, Brazil, 2025.

Variables	n (%)
Age (n = 356)	
Mean (Standard Deviation)	59.4 (14.4)
Median (Minimum-Maximum)	61 (19–96)
Sex (n = 355)	
Male	226 (63.7)
Female	129 (36.3)
Skin color (n = 352)	
White	181 (51.4)
Brown/black	163 (46.3)
Others	8 (2.3)
Level of education (n = 281)	
Elementary education	146 (52.0)
High school	77 (27.4)
Higher education	40 (14.2)
Graduate studies	1 (0.4)
Illiterate	17 (6.0)
Comorbidities (n = 356)	
Yes	304 (85.4)
No	52 (14.6)

NEWS 2 and NEWS A showed high sensitivity to identify ARF. In contrast, MEWS and qSOFA showed specificity (Table 2).

**Table 2:** Sensitivity and specificity of NEWS 2, NEWS A, MEWS, qSOFA scores for identifying acute respiratory failure, shock and cardiorespiratory arrest – São Paulo, SP, Brazil, 2025.

	Acute respiratory failure				Shock				Cardiorespiratory arrest			
	Yes n (%)	No n (%)	SE (%)	S (%)	Yes n (%)	No n (%)	SE (%)	S (%)	Yes n (%)	No n (%)	SE (%)	S (%)
NEWS 2												
High	172 (48.3)	116 (32.6)	88	28	7 (2)	281 (78.9)	88	19	2 (0.6)	286 (80.3)		
Low/medium	23 (6.5)	45 (12.6)			1 (0.3)	67 (18.8)			1 (0.3)	67 (18.8)	67	19
NEWS A												
High	180 (50.6)	133 (37.4)	92	17	8 (2.2)	305 (85.7)	100	12	2 (0.6)	311 (87.4)		
Low/medium	15 (4.2)	28 (7.9)			0 (0)	43 (12.1)			1 (0.3)	42 (11.8)	67	12
MEWS												
High	36 (10.1)	21 (5.9)	19	87	5 (1.4)	52 (14.6)	63	85	0 (0)	57 (16)	0	84
Low	159 (44.7)	140 (39.3)			3 (0.8)	296 (83.1)			3 (0.8)	296 (83.1)		
qSOFA												
High	32 (9)	21 (5.9)	16	87	5 (1.4)	48 (13.5)	62	86	1 (0.3)	52 (14.6)	33	85
Low	163 (45.8)	140 (39.3)			3 (0.8)	300 (84.3)			2 (0.6)	301 (84.6)		

Legend: MEWS – Modified Early Warning Score; qSOFA – quick Sequential Organ Failure Assessment; NEWS2 – National Early Warning Score version 2; NEWS A – National Early Warning Score Age, SE – sensitivity; S – specificity.

Regarding shock identification, NEWS 2 and NEWS A showed high sensitivity, while MEWS and qSOFA showed good specificity (Table 2).

Regarding PCR, NEWS 2 and NEWS A showed medium sensitivity. However, MEWS and qSOFA showed good specificity (Table 2).

Regarding the identification of the need for ICU admission, NEWS 2 and NEWS A presented higher sensitivity values. On the contrary, MEWS and qSOFA presented lower sensitivity (Table 3).

**Table 3:** Sensitivity and specificity of NEWS2, NEWS-A, MEWS, qSOFA scores for identifying Intensive Care Unit admission and death – São Paulo, SP, Brazil, 2025.

		ICU admission						Death				
		Yes n (%)	No n (%)		SE (%)	S (%)		Yes n (%)	No n (%)		SE (%)	S (%)
NEWS 2												
High		157 (44.1)	131 (36.8)		89	27		87 (24.4)	201 (56.5)		87	22
Low/medium		2 (5.6)	48 (13.5)					13 (3.7)	55 (15.4)			
NEWS A												
High		165 (46.3)	148 (41.6)		93	17		92 (25.8)	221 (62.1)		92	14
Low/medium		12 (3.4)	31 (8.7)					8 (2.2)	35 (9.8)			
MEWS												
High		37 (10.4)	20 (5.6)		21	89		20 (5.6)	37 (10.4)		20	86
Low		140 (39.3)	159 (44.7)					80 (22.5)	219 (61.5)			
qSOFA												
High		35 (9.8)	18 (5.1)		20	90		24 (6.7)	29 (8.1)		24	89
Low		142 (39.9)	161 (45.2)					76 (21.3)	227 (63.8)			

Legend: MEWS – Modified Early Warning Score; qSOFA – quick Sequential Organ Failure Assessment; NEWS 2 – National Early Warning Score version 2; NEWS A – National Early Warning Score Age, SE – sensitivity; S – specificity; ICU – Intensive Care Unit.

NEWS 2 and NEWS A have high sensitivity for identifying death. On the other hand, MEWS and qSOFA have superior specificity (Table 3).

A complementary analysis was performed to assess the agreement between clinical deterioration and qSOFA and MEWS, which resulted in a Kappa coefficient of 0.269 (95% CI: 0.21 to 0.4; p-value ≤ 0.001), indicating weak agreement. The agreement between clinical deterioration and NEWS 2 and NEWS A resulted in a Kappa coefficient of 0.677 (95% CI: 0.61 to 0.8; p ≤ 0.001), considered good.

## DISCUSSION

This study investigated the application of EWS in the care of patients with COVID-19. These scores were assessed for their effectiveness in the early identification of clinical deteriorations and their outcomes in a hospital setting. This approach highlights the importance of EWS as tools for monitoring and effective clinical management during the pandemic.

The population consisted mostly of men, with a mean age of 59.4 years, white and with incomplete elementary education. Epidemiological profile studies involving patients hospitalized for COVID-19 indicate a similar mean age^(17)^, intuiting that age is a risk factor for hospitalization, since the elderly population has a decline in the immune system as well as in respiratory, cardiovascular and inflammatory response capacities.

The majority of patients hospitalized for COVID-19 had two or more comorbidities, with hypertension being the most prevalent, followed by diabetes mellitus. COVID-19 has a high lethality rate in individuals over 60 years of age^(18)^, noting that the presence of one or more comorbidities in this population is more prevalent. In a report of 355 deaths from COVID-19 in Italy, the mean age was 79.5 years, and approximately half of the sample (48.5%) had three or more comorbidities, and these factors were determining factors in the fatality rate^(19)^. In another report involving nearly 300,000 cases of COVID-19 in the United States, the hospitalization rate was six times higher and the mortality rate was 12 times higher among those with some comorbidity, when compared to those who did not^(20)^.

Patients with COVID-19 can rapidly progress to severe ARF, shock, and cardiac arrest. In this study, 26.7% of patients progressed to severe respiratory failure, 32.6% to shock, and 27% to cardiac arrest. Therefore, early identification and timely treatment of cases are crucial for a good outcome, as is the use of clinical deterioration scores, such as NEWS 2, at patient admission, demonstrating a high predictive effect for hospitalization and death^(21)^.

EWS, in general, can be part of Rapid Response Systems with the aim of systematizing the early recognition of signs of clinical deterioration and triggering appropriate management^(22)^. Although most studies on the efficiency of EWS in reducing mortality do not have a consistent methodology, their implementation can reduce mortality and unplanned admission to ICUs^(22)^. However, the implementation of EWS in emergency units was recommended and widely used during the COVID-19 pandemic^(23)^.

In our study, NEWS 2 and NEWS A showed good sensitivity, since they identified patients at high risk for clinical deterioration, ICU admission and mortality, but, on the other hand, they had a high degree of false positives. A retrospective analysis published in the United Kingdom showed that NEWS 2 ≥ 5 was very sensitive and not very specific for predicting clinical deterioration^(24)^. In a cohort study aimed at assessing the performance of NEWS and NEWS 2 in relation to the outcome of death and unexpected hospitalization in patients with COVID-19, the scores tested had good performance^(25)^. A multicenter cohort concluded that the predictive capacity of NEWS 2 and variants for serious events and death is questionable^(26)^ and, despite the increase in age, NEWS A has better performance^(27)^.

Contrary to NEWS 2 and NEWS A, both qSOFA and MEWS showed low sensitivity and high specificity, although a retrospective cohort involving pre-hospital vital signs indicated high sensitivity of qSOFA for predicting ICU admission and mortality. According to the authors, qSOFA > 1 represented 58.1 times greater chances of death^(28)^. It is pertinent to consider that both scores do not include oxygen saturation as a variable and that acute respiratory failure was a frequent complication of COVID-19, with its variables being more sensitive to hemodynamic shock, which may justify the finding of this study that NEWS 2 and NEWS A showed better agreement with the outcome of clinical deterioration than MEWS and qSOFA. Another point of observation is the fact that EWS were not developed to predict mortality, but rather clinical deterioration.

When we compare the performance of the scores in this scenario, it seems clear to us that scores that combine respiratory rate and oxygen saturation increase the sensitivity in detecting clinical deterioration in situations where ARF is more frequent. In fact, a publication that compared deterioration scores identified that NEWS 2 was the most sensitive in predicting the severe form of COVID-19 in relation to other scores applied at the bedside^(21)^. A retrospective cohort study conducted in a tertiary hospital in China with 62,403 patients treated in the ES demonstrated that NEWS 2 was significantly better when compared to MEWS and qSOFA in predicting mortality in the ES^(29)^. Therefore, these findings suggest that both NEWS 2 and NEWS A can be an important tool to optimize the nursing assessment process in risk classification and monitoring of patients during their stay in the ES.

Our study was limited by the fact that it was conducted in a single center and, in some cases, by the incomplete recording of clinical parameters for calculating alert scores. However, it was conducted in a referral hospital in the capital of São Paulo, for the care of suspected cases of COVID-19 during the pandemic. Furthermore, this study contributes to the advancement of nursing in improving clinical assessment, capable of quickly identifying signs of clinical deterioration in patients with COVID-19; in evidence-based decision-making, for safer and high-quality care; in standardizing care, ensuring that professionals use uniform criteria for monitoring and intervention; in professional training, increasing the team’s knowledge and ability to deal with critical situations; and in reducing mortality and complications through the effective use of scores, directly benefiting the patient and improving health indicators.

We understand that, given the risks of future pandemics, the topic must remain relevant, as it deals closely with how nursing professionals will organize themselves to provide a rapid and assertive response. In this context, we believe it is rational to use the best tools, which are easy to apply and low cost, to improve the performance of detecting clinical deterioration and increase health safety.

## CONCLUSION

In this context, both NEWS 2 and NEWS A stand out among EWS and can be useful in supporting nurses in patient assessment, since they have high sensitivity, quickly and objectively identifying possible clinical deteriorations and enabling the early implementation of measures in the care of patients admitted to an ES. In contrast, MEWS and qSOFA should be prioritized for the confirmation of negative results in non- patients, since they have greater specificity in verifying clinical deterioration in patients.

## Data Availability

The entire data set supporting the results of this study was published in the article itself.
