# Supplementary material for: Assessment of the performance of early warning scores in patients with COVID-19
Source: Rev Esc Enferm USP. 2025 May 26;59:e20240371. doi: 10.1590/1980-220X-REEUSP-2024-0371en (PMC12118358; doi:10.1590/1980-220X-REEUSP-2024-0371en)
Supplement: Translation

# Translation: Avaliação do desempenho dos escores de alerta precoce em pacientes com COVID-19

**DOI:** 10.1590/1980-220X-REEUSP-2024-0371pt

**Authors:** Victoria Castilho Bartolomeu, Luiz Humberto Vieri Piacezzi, Karina Aparecida Lopes da Costa, Ruth Ester Assayag Batista, Cássia Regina Vancini Campanharo, Maria Carolina Barbosa Teixeira Lopes

**Affiliations:** 1 Universidade Federal de São Paulo, Escola Paulista de Enfermagem, São Paulo, SP, Brasil.; 2 Universidade Federal de São Paulo, Escola Paulista de Enfermagem, Departamento de Enfermagem Clínica e Cirúrgica, São Paulo, SP, Brasil.

**Keywords:** Triagem, Escore de Alerta Precoce, COVID-19, Serviços Médicos de Emergência, Cuidados de Enfermagem

## Abstract

**Objetivo:**

Avaliar o desempenho dos escores de alerta precoce *Modified Early Warning Score, National Early Warning Score 2, National Early Warning Score Age* e *quick Sepsis Related Organ Failure Assessment* para identificação das deteriorações clínicas em pacientes com COVID-19.

**Método:**

Estudo de coorte retrospectivo. Incluíram-se pacientes com diagnóstico confirmado de COVID-19. As deteriorações clínicas foram insuficiência respiratória aguda, choque e parada cardiorrespiratória, e os desfechos foram internação não programada na Unidade de Terapia Intensiva, alta e óbito. Foram determinadas a sensibilidade e probabilidade de resultado positivo nos doentes (verdadeiro positivo), e especificidade na probabilidade de resultado negativo em não doentes (verdadeiro negativo).

**Resultados:**

O *National Early Warning Score 2* e o *National Early Warning Score Age* apresentaram melhores valores de sensibilidade para identificação precoce das deteriorações e dos desfechos.

**Conclusão:**

O NEWS 2 e o NEWS A apresentaram melhor sensibilidade e especificidade, recomendando-se seu uso para subsidiar a avaliação clínica do paciente com COVID-19. Este estudo contribui para a prática assistencial, ao avaliar escores que podem aprimorar a avaliação clínica, permitir a identificação rápida dos sinais de deterioração dos pacientes acometidos pela COVID-19 e intervenções precoces.

## INTRODUÇÃO

Os serviços de emergência (SEs) desempenham um papel fundamental no acesso ao Sistema Único de Saúde, e são um dos principais meios de acesso aos cuidados em saúde da população brasileira^(1)^. Mundialmente, há um aumento da procura pelos SEs de forma que esse não é um fenômeno novo, e suas causas são multifatoriais, como o aumento da expectativa de vida e da incidência das doenças crônicas não transmissíveis, pobreza e violência urbana, além da possibilidade de rápido acesso à medicina diagnóstica e tratamentos complexos em um único lugar^(2)^.

No contexto do SE, o enfermeiro atua como protagonista para a observação clínica e detecção precoce da piora clínica, especialmente no cenário de pandemia, no qual o quadro clínico poderia deteriorar rapidamente. Assim, com o apoio dos escores de alerta precoce (EAPs), é possível melhorar a segurança em saúde e, consequentemente, os indicadores hospitalares^(2)^.

A pandemia de COVID-19 aumentou a pressão sobre os SEs que passaram a receber aproximadamente 10,3% mais usuários, quando comparado aos anos anteriores^(3)^. Em resposta, os centros de saúde expandiram sua oferta de leitos, recursos materiais e humanos, incentivando ou implantando fluxos que aumentassem a vigilância e segurança dos pacientes admitidos^(4)^, como os EAPs, que começaram a ser utilizados nesse contexto^(5)^.

Os EAPs são modelos que atribuem pontuações de acordo com variáveis fisiológicas aferidas à beira-leito, estratificando o risco de deterioração clínica, o que possibilita acionar atendimento de saúde precocemente^(6)^. Com o tempo, modificações e adaptações realizadas nesses modelos deram origem a diversos tipos de EAPs, validados em cenários distintos^(7)^. Estudos demonstram que a utilização dos escores de identificação precoce de deterioração clínica subsidiam a avaliação clínica de forma mais assertiva, propiciando a comunicação padronizada sobre o estado de saúde e diminuindo a mortalidade^(8)^.

Diante dos diversos escores elaborados e adaptados, é imprescindível a identificação do EAP mais preciso para uso na prática do rastreio de deteriorações clínicas, uma vez que a COVID-19, frequentemente, causa “hipoxemia silenciosa”, evento no qual há pouca alteração nos outros parâmetros fisiológicos^(9)^. Para tanto, este estudo objetivou avaliar a performance do *Modified Early Warning Score* (MEWS), *quick Sequential Organ Failure Assessment* (qSOFA), *National Early Warning Score version 2* (NEWS 2) e *National Early Warning Score Age* (NEWS A) em relação à capacidade de identificar a deterioração clínica precoce (insuficiência respiratória aguda, choque e parada cardiorrespiratória), internação em Unidade de Terapia Intensiva (UTI), e desfecho alta e óbito em pacientes internados no SE com o diagnóstico de COVID-19. Este estudo contribui para a realização do cuidado de enfermagem baseado em evidências, a medida em que demonstra como os escores são ferramentas de apoio para a tomada de decisão clínica do enfermeiro na vigilância de pacientes internados, especialmente em ambientes e dinâmicos como os SEs, contribuindo para a diminuição dos agravos em saúde e da mortalidade.

## MÉTODO

### Desenho de Estudo, Período e Local do Estudo

Estudo de coorte retrospectivo, cujos passos metodológicos foram norteados pela ferramenta *STrengthening the Reporting of OBservational studies in Epidemiology* (STROBE), realizado em um hospital universitário de grande porte^(10)^ entre julho de 2022 e maio de 2023. O SE, durante a pandemia de COVID-19, instituiu uma sala de classificação de risco específica para pacientes com sintomas respiratórios e uma unidade de insuficiência respiratória para casos suspeitos ou confirmados de COVID-19 que necessitavam de internação.

### População

Foram utilizados dados secundários dos pacientes maiores de 18 anos, admitidos no SE com diagnóstico confirmado de COVID-19, por meio do exame de *Reverse Transcription Polymerase Chain Reaction*, entre abril e agosto de 2020, que aceitaram participar do estudo. Foram excluídos os pacientes que apresentavam dados incompletos para o cálculo dos escores e gestantes.

### Coleta de Dados

Os dados foram obtidos por meio da análise dos prontuários eletrônicos e compostos por variáveis sociodemográficas (idade, sexo, cor da pele e escolaridade), comorbidades, sinais e sintomas, categoria da classificação de risco, sinais vitais para cálculo dos escores no momento da admissão, ocorrência de deterioração clínica, marcadores de gravidade, e desfechos (alta e óbito).

Neste estudo, considerou-se como deterioração clínica: (1) insuficiência respiratória aguda (IRpA), caracterizada pela introdução ou aumento da titulação de oxigênio ou escalonamento na modalidade ventilatória; (2) choque, definido pela introdução de uma nova droga vasoativa; e (3) parada cardiorrespiratória (PCR).

Os EAPs avaliados foram MEWS, qSOFA, NEWS 2 e NEWS A.

O MEWS pontua de 1 a 3 (sendo 1 a pontuação esperada para padrões dentro da normalidade, e 3, a pontuação para padrões alterados) os seguintes parâmetros: frequência cardíaca (FC), sendo 0: 51–100 bpm, 1: 101–110 bpm, 2: 111–130 bpm e 3: > 130 bpm ou < 40 bpm; frequência respiratória (FR) sendo 0: 12–20 ipm, 1: 21–24 ipm, 2: 25-29 ipm e 3: > 30 ipm; temperatura (T), sendo 0: 36,1 ºC–38 ºC, 1: < 36 ºC ou > 38 ºC e 2: ≥ 39,1 ºC; pressão arterial sistólica (PAS), sendo 0: > 100 mmHg, 1: 81–100 mmHg, 2: 71–80 mmHg e 3: < 70 mmHg; e nível de consciência, de acordo com a *Glasgow Coma Scale* (GCS), sendo 0: GCS 15, 1: GCS 14–13, 2: GCS 12–9 e 3: GCS < 9. A soma da pontuação varia de 0 a 14, e caracterizamos risco clínico alto ≥ 4 pontos^(11)^, uma vez que esse resultado tende a apontar para risco de choque, insuficiência respiratória, falência cardiovascular, acidose metabólica, hipóxia e deterioração neurológica^(11)^, exigindo monitoramento mais rigoroso e intervenção da equipe.

O qSOFA, escore amplamente utilizado para o rastreio de sepse, avalia FR, sendo 1 ponto para FR > 22 ipm, PAS, sendo 1 ponto para PAS ≤ 100 mmHg, e alteração no nível de consciência, sendo 1 ponto para desorientação ou diminuição da resposta a estímulos. A soma da pontuação varia de 0 a 3, e considera-se baixo risco para sepse de 0-1 ponto e alto risco de 2-3 pontos^(12)^, havendo a necessidade de avaliação com urgência e intervenções imediatas.

O NEWS 2 pode variar de 0 a 20 pontos, com uma pontuação mais alta, indicando maior risco de deterioração clínica, e é composto pelos seguintes parâmetros: FR (12–≥ 30 ipm); FC (51–>130 bpm); saturação de oxigênio (≥ 96%–≤ 91%); necessidade de suporte de oxigênio adicional; PAS (≥ 100 mmHg–≤ 80 mmHg); e nível de consciência (GCS 15–< 9)^(13)^. O NEWS A utiliza os mesmos parâmetros do NEWS 2,somando-se 3 pontos para pacientes com 65 anos ou mais^(14)^. Categorizamos o risco clínico como baixo risco (0–4), risco moderado (5–6) e alto risco (≥ 7), conforme preconiza o *Royal College of Physicians* ^(15)^. Indivíduos com baixo risco, mas que receberam 3 pontos em alguma categoria, foram considerados risco moderado^(15)^.

### Análise dos Resultados e Estatística

Os dados foram armazenados na plataforma *Research Electronic Data Capture* (REDCap)^®^. Para as variáveis contínuas, calcularam-se a média, desvio padrão, mediana, mínimo e máximo, e, para as categóricas, calcularam-se frequência e percentual. O desempenho dos escores foi calculado por meio da sensibilidade, que é a probabilidade de um resultado positivo nos doentes (verdadeiro positivo), e especificidade, que é a probabilidade de um resultado negativo em não doentes (verdadeiro negativo).

Realizada análise complementar por meio do coeficiente de concordância Kappa, interpretado de acordo com Altman^(16)^ por pobre (<0,2), razoável (0,21 a 0,4), moderada (0,41 a 0,6), boa (0,61 a 0,8) e muito boa (0,8 a 1). Foram calculados o Kappa de Fleiss, para analisar a concordância entre os quatro escores e a deterioração clínica, e o Kappa de Cohen, entre os escores individualmente.

### Aspectos Éticos

O estudo foi norteado pela Resolução n° 466/2012, sendo aprovado pelo Comitê de Ética em Pesquisa da Universidade Federal de São Paulo, sob Parecer n° 4.087.059. O Termo de Consentimento Livre e Esclarecido foi obtido online.

## RESULTADOS

Foram incluídos 356 pacientes, sendo a maioria homens, brancos, com ensino fundamental incompleto e com comorbidades (Tabela 1). A hipertensão arterial sistêmica foi a comorbidade mais frequente (n = 211, 59,3%), seguida de diabetes mellitus (n = 137, 38,5%), cardiopatias (n = 76, 21,3%) e transplante de órgãos sólidos (n = 70, 19,7%).

**Tabela 1:** Variáveis sociodemográficas e presença de comorbidades da população estudada – São Paulo, SP, Brasil, 2025.

Variáveis	n (%)
Idade (n = 356)	
Média (Desvio Padrão)	59,4 (14,4)
Mediana (Mínimo–Máximo)	61 (19–96)
Sexo (n = 355)	
Masculino	226 (63,7)
Feminino	129 (36,3)
Cor da pele (n = 352)	
Branco	181 (51,4)
Pardo/preto	163 (46,3)
Outros	8 (2,3)
Grau de instrução (n = 281)	
Ensino fundamental	146 (52,0)
Ensino médio	77 (27,4)
Ensino superior	40 (14,2)
Pós-graduação	1 (0,4)
Não alfabetizado	17 (6,0)
Comorbidades (n = 356)	
Sim	304 (85,4)
Não	52 (14,6)

O NEWS 2 e o NEWS A apresentaram alta sensibilidade para identificar a IRpA. Em contrapartida, o MEWS e o qSOFA apresentam especificidade (Tabela 2).

**Tabela 2:** Sensibilidade e especificidade dos escores NEWS 2, NEWS A, MEWS, qSOFA para identificação de insuficiência respiratória aguda, choque e parada cardiorrespiratória – São Paulo, SP, Brasil, 2025.

	Insuficiência respiratória aguda				Choque				Parada cardiorrespiratória			
	Sim n (%)	Não n (%)	S (%)	E (%)	Sim n (%)	Não n (%)	S (%)	E (%)	Sim n (%)	Não n (%)	S (%)	E (%)
NEWS 2												
Alto	172 (48,3)	116 (32,6)	88	28	7 (2)	281 (78,9)	88	19	2 (0,6)	286 (80,3)		
Baixo/médio	23 (6,5)	45 (12,6)			1 (0,3)	67 (18,8)			1 (0,3)	67 (18,8)	67	19
NEWS A												
Alto	180 (50,6)	133 (37,4)	92	17	8 (2,2)	305 (85,7)	100	12	2 (0,6)	311 (87,4)		
Baixo/médio	15 (4,2)	28 (7,9)			0 (0)	43 (12,1)			1 (0,3)	42 (11,8)	67	12
MEWS												
Alto	36 (10,1)	21 (5,9)	19	87	5 (1,4)	52 (14,6)	63	85	0 (0)	57 (16)	0	84
Baixo	159 (44,7)	140 (39,3)			3 (0,8)	296 (83,1)			3 (0,8)	296 (83,1)		
qSOFA												
Alto	32 (9)	21 (5,9)	16	87	5 (1,4)	48 (13,5)	62	86	1 (0,3)	52 (14,6)	33	85
Baixo	163 (45,8)	140 (39,3)			3 (0,8)	300 (84,3)			2 (0,6)	301 (84,6)		

Legenda: MEWS – *Modified Early Warning Score*; qSOFA – *quick Sequential Organ Failure Assessment*; NEWS2 – *National Early Warning Score version 2*; NEWS A – *National Early Warning Score Age*; S – sensibilidade; E – especificidade.

Em relação à identificação do choque, o NEWS 2 e NEWS A apresentaram alta sensibilidade. Já o MEWS e qSOFA apresentaram boa especificidade (Tabela 2).

Em relação à PCR, o NEWS 2 e NEWS A apresentaram sensibilidade mediana. Entretanto, o MEWS e qSOFA apresentaram boa especificidade (Tabela 2).

Em relação à identificação de necessidade de internação na UTI, o NEWS 2 e NEWS A apresentaram maiores valores de sensibilidade. Ao contrário, o MEWS e qSOFA apresentam sensibilidade inferior (Tabela 3).

**Tabela 3:** Sensibilidade e especificidade dos escores NEWS2, NEWS-A, MEWS, qSOFA para identificação de internação na Unidade de Terapia Intensiva e óbito – São Paulo, SP, Brasil, 2025.

		Internação na UTI						Óbito				
		Sim n (%)	Não n (%)		S (%)	E (%)		Sim n (%)	Não n (%)		S (%)	E (%)
NEWS 2												
Alto		157 (44,1)	131 (36,8)		89	27		87 (24,4)	201 (56,5)		87	22
Baixo/Médio		2 (5,6)	48 (13,5)					13 (3,7)	55 (15,4)			
NEWS A												
Alto		165 (46,3)	148 (41,6)		93	17		92 (25,8)	221 (62,1)		92	14
Baixo/Médio		12 (3,4)	31 (8,7)					8 (2,2)	35 (9,8)			
MEWS												
Alto		37 (10,4)	20 (5,6)		21	89		20 (5,6)	37 (10,4)		20	86
Baixo		140 (39,3)	159 (44,7)					80 (22,5)	219 (61,5)			
qSOFA												
Alto		35 (9,8)	18 (5,1)		20	90		24 (6,7)	29 (8,1)		24	89
Baixo		142 (39,9)	161 (45,2)					76 (21,3)	227 (63,8)			

Legenda: MEWS – *Modified Early Warning Score*; qSOFA – *quick Sequential Organ Failure Assessment*; NEWS 2 – *National Early Warning Score version 2*; NEWS A – *National Early Warning Score Age*; S – sensibilidade; E – especificidade; UTI – Unidade de Terapia Intensiva.

O NEWS 2 e NEWS A apresentam alta sensibilidade para identificar óbito. De outro modo, o MEWS e qSOFA apresentam especificidade superior (Tabela 3).

Foi realizada uma análise complementar para avaliar a concordância entre a deterioração clínica e o qSOFA e MEWS, que resultou em um coeficiente Kappa de 0,269 (IC 95%: 0,21 a 0,4; p-valor ≤ 0,001), indicando concordância fraca. A concordância entre a deterioração clínica e o NEWS 2 e NEWS A resultou em um coeficiente Kappa de 0,677 (IC:95%: 0,61 a 0,8; p ≤ 0,001), considerada boa.

## DISCUSSÃO

Este estudo investigou a aplicação dos EAPs no cuidado de pacientes com COVID-19. Esses escores foram avaliados quanto à sua eficácia na identificação precoce de deteriorações clínicas e seus desfechos em um ambiente hospitalar. Essa abordagem destaca a importância dos EAPs como ferramentas para o monitoramento e manejo clínico eficaz durante a pandemia.

A população foi constituída em sua maioria por homens, com média de idade 59,4 anos, brancos e com ensino fundamental incompleto. Estudos de perfil epidemiológico, envolvendo pacientes internados por COVID-19, apontam para média de idade semelhante^(17)^, intuindo que a idade é um fator de risco para internação hospitalar, uma vez que a população de idade avançada apresenta declínio do sistema imunológico, bem como das capacidades respiratória, cardiovascular e de resposta inflamatória.

A maioria dos pacientes internados por COVID-19 tinha duas ou mais comorbidades, sendo hipertensão arterial sistêmica a mais prevalente, seguida de diabetes mellitus. A COVID-19 apresenta alta letalidade em indivíduos acima dos 60 anos^(18)^, notando-se que a presença de uma ou mais comorbidades nesta população é mais prevalente. Em um relatório de 355 óbitos por COVID-19 na Itália, a média de idade foi 79,5 anos, sendo que, aproximadamente, metade da amostra (48,5%) apresentava três ou mais comorbidades, e esses fatores foram determinantes na taxa de letalidade^(19)^. Em outro relatório envolvendo quase 300 mil casos de COVID-19 nos Estados Unidos, a taxa de hospitalização foi seis vezes maior, e a de mortalidade, 12 vezes maior entre os que tinham alguma comorbidade, quando comparados aos que não tinham^(20)^.

Pacientes com COVID-19 podem evoluir rapidamente para IRpA grave, choque e PCR. Neste estudo, 26,7% dos pacientes evoluíram para insuficiência respiratória grave, 32,6%, para choque, e 27%, para PCR. Desse modo, a identificação precoce e o tratamento oportuno dos casos são cruciais para um bom desfecho, assim como a utilização de escores de deterioração clínica, como o NEWS 2, na admissão do paciente, demonstrando um alto efeito preditivo para internação e óbito^(21)^.

Os EAPs, em geral, podem compor Sistemas de Resposta Rápida com o objetivo de sistematizar o reconhecimento precoce dos sinais de deterioração clínica e acionar o manejo adequado^(22)^. Apesar de a maioria dos estudos sobre a eficiência dos EAPs na redução da mortalidade não terem metodologia consistente, a sua implementação pode reduzir a mortalidade e admissão não planejada em UTIs^(22)^. No entanto, a implantação de EAPs em unidade de emergência foi recomendada e amplamente utilizada durante a pandemia de COVID-19^(23)^.

Em nosso estudo, o NEWS 2 e NEWS A apresentaram boa sensibilidade, uma vez que identificaram os pacientes de alto risco para deterioração clínica, internação em UTI e mortalidade, mas, por outro lado, tinham alto grau de falsos positivos. Uma análise retrospectiva publicada no Reino Unido mostrou que o NEWS 2 ≥ 5 foi muito sensível e pouco específico para prever deterioração clínica^(24)^. Em estudo de coorte com o objetivo de avaliar o desempenho do NEWS e NEWS 2 em relação ao desfecho de óbito e internação inesperada em pacientes com COVID-19, os escores testados tiveram boa performance^(25)^. Coorte multicêntrica concluiu que a capacidade preditiva de evento grave e óbito do NEWS 2 e variantes são questionáveis^(26)^ e, apesar do acréscimo da idade, o NEWS A apresenta melhor desempenho^(27)^.

Na contramão do NEWS 2 e NEWS A, tanto o qSOFA quanto o MEWS mostraram baixa sensibilidade e alta especificidade, embora coorte retrospectiva envolvendo sinais vitais pré-hospitalares apontou alta sensibilidade do qSOFA para a predição de internação em UTI e mortalidade. Segundo os autores, qSOFA>1 representava 58,1 vezes mais chances de óbito^(28)^. É pertinente ponderar que ambos escores não contemplam a saturação de oxigênio como variável e a insuficiência respiratória aguda era complicação frequente da COVID-19, sendo suas variáveis mais sensíveis ao choque hemodinâmico, o que pode justificar o achado deste estudo de que o NEWS 2 e NEWS A apresentaram melhor concordância com o desfecho de deterioração clínica do que o MEWS e qSOFA. Um outro ponto de observação é o fato de que os EAPs não foram desenvolvidos para prever mortalidade, mas sim deterioração clínica.

Quando comparamos os desempenhos dos escores neste cenário, parece-nos claro que escores que contam com combinação de frequência respiratória e saturação de oxigênio aumentam a sensibilidade na detecção da deterioração clínica em situações em que a IRpA é mais frequente. De fato, publicação que comparou escores de deterioração identificou que o NEWS 2 foi o mais sensível para prever a forma grave da COVID-19 em relação a outros escores aplicados à beira do leito^(21)^. Estudo de coorte retrospectivo, realizado em um hospital terciário na China, com 62.403 pacientes atendidos no SE, demonstrou que o NEWS 2 foi, significativamente, melhor quando comparado ao MEWS e ao qSOFA para prever mortalidade no SE^(29)^. Portanto, esses achados sugerem que tanto o NEWS 2 quanto o NEWS A podem ser uma importante ferramenta para otimizar o processo de avaliação do enfermeiro na classificação de risco e no monitoramento dos pacientes durante sua permanência no SE.

Nosso estudo teve como limitação a realização em centro único e, em alguns casos, a incompletude dos registros dos parâmetros clínicos para cálculo dos escores de alerta. Entretanto, foi realizado em um hospital de referência, na capital paulista, para o atendimento dos casos suspeitos de COVID-19 durante a pandemia. Além disso, este estudo contribui para o avanço da enfermagem no aprimoramento da avaliação clínica, capaz de identificar rapidamente os sinais de deterioração clínica de pacientes com COVID-19; na tomada de decisão baseada em evidências, para um cuidado mais seguro e de alta qualidade; na padronização dos cuidados, garantindo que os profissionais utilizem critérios uniformes para monitoramento e intervenção; na capacitação profissional, aumentando o conhecimento e a habilidade da equipe para lidar com situações críticas; e na redução da mortalidade e das complicações por meio do uso eficaz dos escores, beneficiando diretamente o paciente e melhorando os indicadores de saúde.

Entendemos que, mediante os riscos de futuras pandemias, a temática deve se manter relevante, pois trata intimamente de como os profissionais de enfermagem irão se organizar para que haja uma resposta rápida e assertiva. Neste contexto, julgamos racional usar as melhores ferramentas, facilmente aplicáveis e de baixo custo, que melhorem o desempenho da detecção da deterioração clínica e aumentem a segurança em saúde.

## CONCLUSÃO

Nesse contexto, tanto o NEWS 2 quanto o NEWS A se destacam entre os EAPs, e podem ser úteis para subsidiar os enfermeiros na avaliação do paciente, uma vez que apresentam alta sensibilidade, identificando de maneira rápida e objetiva possíveis deteriorações clínicas e possibilitando a implementação precoce de medidas na assistência ao paciente internado em um SE. Em contrapartida, o MEWS e qSOFA devem ser priorizados para a confirmação de resultados negativos em não doentes, uma vez que apresentam maior especificidade na verificação de deterioração clínica dos pacientes.

## Data Availability

Todo o conjunto de dados que dá suporte aos resultados deste estudo foi publicado no próprio artigo.

# Translation: Evaluación del desempeño de puntajes de alerta temprana en pacientes con COVID-19

**DOI:** 10.1590/1980-220X-REEUSP-2024-0371es

**Authors:** Victoria Castilho Bartolomeu, Luiz Humberto Vieri Piacezzi, Karina Aparecida Lopes da Costa, Ruth Ester Assayag Batista, Cássia Regina Vancini Campanharo, Maria Carolina Barbosa Teixeira Lopes

**Affiliations:** 1 Universidade Federal de São Paulo, Escola Paulista de Enfermagem, São Paulo, SP, Brasil.; 2 Universidade Federal de São Paulo, Escola Paulista de Enfermagem, Departamento de Enfermagem Clínica e Cirúrgica, São Paulo, SP, Brasil.

**Keywords:** Triaje, Puntuación de Alerta Temprana, COVID-19, Servicios Médicos de Urgencia, Atención de Enfermería

## Abstract

**Objetivo:**

Evaluar el desempeño de las puntuaciones de alerta temprana *Modified Early Warning Score, National Early Warning Score 2, National Early Warning Score Age y quick Sepsis Related Organ Failure Assessment* para identificar deterioros clínicos en pacientes con COVID-19.

**Método:**

Estudio de cohorte retrospectivo. Se incluyeron pacientes con diagnóstico confirmado de COVID-19. Los deterioros clínicos fueron insuficiencia respiratoria aguda, shock y paro cardiorrespiratorio, y los resultados fueron ingreso no programado a la Unidad de Cuidados Intensivos, alta y muerte. Se determinó la sensibilidad y probabilidad de un resultado positivo en pacientes (verdadero positivo) y la especificidad en la probabilidad de un resultado negativo en no pacientes (verdadero negativo).

**Resultados:**

*El National Early Warning Score 2* y el *National Early Warning Score Age* presentaron mejores valores de sensibilidad para la identificación temprana de deterioros y desenlaces.

**Conclusión:**

NEWS 2 y NEWS A mostraron mejor sensibilidad y especificidad, recomendando su uso para apoyar la evaluación clínica de pacientes con COVID-19. Este estudio contribuye a la práctica asistencial, al evaluar puntuaciones que pueden mejorar la evaluación clínica, permitir una rápida identificación de signos de deterioro en pacientes afectados por COVID-19 e intervenciones tempranas.

## INTRODUCCIÓN

Los servicios de emergencia (SE) desempeñan un papel fundamental en el acceso al Sistema Único de Salud, y son una de las principales vías de acceso a la atención sanitaria de la población brasileña^(1)^. En todo el mundo, ha habido un aumento en la demanda de los SE, lo que no es un fenómeno nuevo, y sus causas son multifactoriales, como el aumento de la esperanza de vida y la incidencia de enfermedades crónicas no transmisibles, la pobreza y la violencia urbana, así como la posibilidad de acceso rápido a la medicina de diagnóstico y tratamientos complejos en un solo lugar^(2)^.

En el contexto de los SE, las enfermeras desempeñan un papel clave en la observación clínica y la detección precoz del empeoramiento clínico, especialmente en un escenario pandémico, en el que el cuadro clínico podría deteriorarse rápidamente. Así, con el apoyo de las puntuaciones de alerta temprana (PAT), es posible mejorar la seguridad en salud y, en consecuencia, los indicadores hospitalarios^(2)^.

La pandemia de COVID-19 ha aumentado la presión sobre los centros de salud, que han recibido aproximadamente un 10,3% más de usuarios que en años anteriores^(3)^. En respuesta, los centros sanitarios ampliaron su oferta de camas, recursos materiales y humanos, fomentando o implantando flujos que aumentaran la vigilancia y la seguridad de los pacientes ingresados^(4)^, como las PAT, que empezaron a utilizarse en este contexto^(5)^.

Las PATs son modelos que asignan puntuaciones en función de variables fisiológicas medidas a pie de cama, estratificando el riesgo de deterioro clínico, lo que permite activar precozmente la asistencia sanitaria^(6)^. Con el tiempo, las modificaciones y adaptaciones de estos modelos han dado lugar a distintos tipos de PATs, validados en diferentes escenarios^(7)^. Los estudios han demostrado que el uso de puntuaciones para la identificación precoz del deterioro clínico subvenciona la evaluación clínica de forma más asertiva, proporcionando una comunicación estandarizada sobre el estado de salud y reduciendo la mortalidad^(8)^.

Dadas las diversas puntuaciones que se han desarrollado y adaptado, es esencial identificar la PAT más precisa para su uso en el cribado del deterioro clínico, ya que la COVID-19 suele causar «hipoxemia silenciosa», un evento en el que apenas se producen cambios en otros parámetros fisiológicos^(9)^. Para ello, este estudio tuvo como objetivo evaluar el rendimiento del *Modified Early Warning Score* (MEWS), *quick Sequential Organ Failure Assessment* (qSOFA), *National Early Warning Score versión 2* (NEWS 2) y *National Early Warning Score Age* (NEWS A) en relación a su capacidad para identificar el deterioro clínico precoz (insuficiencia respiratoria aguda, shock y parada cardiorrespiratoria), el ingreso en la Unidad de Cuidados Intensivos (UCI), y el alta y la muerte en pacientes ingresados en urgencias con diagnóstico de COVID-19. Este estudio contribuye a los cuidados de enfermería basados en la evidencia al demostrar cómo las puntuaciones son herramientas de apoyo para la toma de decisiones clínicas de las enfermeras en la vigilancia de los pacientes hospitalizados, especialmente en entornos dinámicos como los SUH, contribuyendo a la reducción de los problemas de salud y la mortalidad.

## MÉTODO

### Diseño del Estudio, Periodo y Localización

Se trata de un estudio de cohortes retrospectivo, cuyos pasos metodológicos se guiaron por la herramienta Strengthening *the Reporting of Observational Studies in Epidemiology* (STROBE), realizado en un gran hospital universitario^(10)^ entre julio de 2022 y mayo de 2023. Durante la pandemia de COVID-19, el SE estableció una sala de clasificación de riesgo específica para pacientes con síntomas respiratorios y una unidad de insuficiencia respiratoria para casos sospechosos o confirmados de COVID-19 que requirieran hospitalización.

### Población

Se utilizaron datos secundarios de pacientes mayores de 18 años que ingresaron en urgencias con diagnóstico confirmado de COVID-19 mediante la prueba *de Reverse Transcription Polymerase Chain Reaction (Reacción en Cadena de la Polimerasa de Transcripción Inversa)* entre abril y agosto de 2020 y que aceptaron participar en el estudio. Se excluyeron los pacientes con datos incompletos para el cálculo de las puntuaciones y las mujeres embarazadas.

### Recolección de Datos

Los datos se obtuvieron mediante el análisis de las historias clínicas electrónicas y consistieron en variables sociodemográficas (edad, sexo, color de piel y escolaridad), comorbilidades, signos y síntomas, categoría de clasificación de riesgo, signos vitales para calcular las puntuaciones al ingreso, aparición de deterioro clínico, marcadores de gravedad y resultados (alta y muerte).

En este estudio, se consideró deterioro clínico: (1) la insuficiencia respiratoria aguda (IRA), caracterizada por la introducción o el aumento de la titulación de oxígeno o la intensificación de la modalidad ventilatoria; (2) el shock, definido por la introducción de un nuevo fármaco vasoactivo; y (3) la parada cardiorrespiratoria (PCR). Los PAT evaluados fueron MEWS, qSOFA, NEWS 2 y NEWS A.

MEWS puntúa los siguientes parámetros de 1 a 3 (siendo 1 la puntuación esperada para patrones normales y 3 para patrones alterados): frecuencia cardíaca (FC), 0: 51–100 lpm, 1: 101–110 lpm, 2: 111–130 lpm y 3: > 130 lpm o < 40 lpm; frecuencia respiratoria (FR), 0: 12–20 ipm, 1: 21-24 ipm, 2: 25–29 ipm y 3: > 30 ipm; temperatura (T), donde 0: 36,1 ºC–38 ºC, 1: < 36 ºC o > 38 ºC y 2: ≥ 39,1 ºC; presión arterial sistólica (PAS), donde 0: > 100 mmHg, 1: 81–100 mmHg, 2: 71–80 mmHg y 3: < 70 mmHg; nivel de consciencia, según la *Glasgow Coma Scale (Escala de Coma de Glasgow)* (GCS), con 0: GCS 15, 1: GCS 14–13, 2: GCS 12–9 y 3: GCS < 9. La suma de las puntuaciones oscila entre 0 y 14, y caracterizamos el riesgo clínico alto como ≥ 4 puntos^(11)^, ya que esta puntuación tiende a indicar riesgo de shock, insuficiencia respiratoria, insuficiencia cardiovascular, acidosis metabólica, hipoxia y deterioro neurológico^(11)^, requiriendo una monitorización e intervención más rigurosa por parte del equipo.

El qSOFA, una puntuación ampliamente utilizada para el cribado de la sepsis, evalúa el RR, con 1 punto para RR > 22 ipm, la PAS, 1 punto por PAS ≤ 100 mmHg, y la alteración del nivel de conciencia, 1 punto por desorientación o disminución de la respuesta a estímulos. La suma de las puntuaciones oscila entre 0 y 3 y se considera de bajo riesgo de sepsis con 0-1 punto y de alto riesgo con 2-3 puntos^(12)^, lo que requiere una evaluación urgente e intervenciones inmediatas.

La NEWS 2 puede oscilar entre 0 y 20 puntos, indicando una puntuación más alta un mayor riesgo de deterioro clínico, y se compone de los siguientes parámetros: RR (12 – ≥ 30 ipm); FC (51 – > 130 lpm); saturación de oxígeno (≥ 96% – ≤ 91%); necesidad de soporte adicional de oxígeno; PAS (≥ 100 mmHg –≤ 80 mmHg); y nivel de consciencia (GCS 15 – < 9)^(13)^. NEWS A utiliza los mismos parámetros que NEWS 2, añadiendo 3 puntos para los pacientes de 65 años o más^(14)^. Se categorizó el riesgo clínico como riesgo bajo (0–4), riesgo moderado (5–6) y riesgo alto (≥ 7), tal y como recomienda el *Royal College of Physicians* ^(15)^. Los individuos con riesgo bajo, pero que recibieron 3 puntos en cualquier categoría, se consideraron de riesgo moderado^(15)^.

### Análisis de los Resultados y Estadísticas

Los datos se almacenaron en la plataforma *Research Electronic Data Capture* (REDCap)^®^. Para las variables continuas, se calcularon la media, la desviación estándar, la mediana, el mínimo y el máximo, y para las variables categóricas, la frecuencia y el porcentaje. El rendimiento de las puntuaciones se calculó utilizando la sensibilidad, que es la probabilidad de un resultado positivo en pacientes (verdadero positivo), y la especificidad, que es la probabilidad de un resultado negativo en no pacientes (verdadero negativo).

Se realizó un análisis complementario utilizando el coeficiente de concordancia Kappa, interpretado según Altman^(16)^ como deficiente (< 0,2), razonable (0,21 a 0,4), moderado (0,41 a 0,6), bueno (0,61 a 0,8) y muy bueno (0,8 a 1). Se calculó el Kappa de Fleiss para analizar la concordancia entre las cuatro puntuaciones y el deterioro clínico, y el Kappa de Cohen entre las puntuaciones individualmente.

### Aspectos Éticos

El estudio fue orientado por la Resolución 466/2012 y aprobado por el Comité de Ética en Investigación de la Universidad Federal de São Paulo, bajo el Informe nº 4.087.059. El formulario de consentimiento informado se obtuvo en línea.

## RESULTADOS

Fueron incluidos 356 pacientes, en su mayoría hombres, de raza blanca, con educación primaria incompleta y comorbilidades (Tabla 1). La hipertensión arterial sistémica fue la comorbilidad más frecuente (n = 211, 59,3%), seguida de la diabetes mellitus (n = 137, 38,5%), la cardiopatía (n = 76, 21,3%) y el trasplante de órgano sólido (n = 70, 19,7%).

**Tabla 1:** Variables sociodemográficas y presencia de comorbilidades en la población de estudio – São Paulo, SP, Brasil, 2025.

Variables	n (%)
Edad (n = 356)	
Media (Desvío Estándar)	59,4 (14,4)
Mediana (Mínimo–Máximo)	61 (19–96)
Sexo (n = 355)	
Masculino	226 (63,7)
Femenino	129 (36,3)
Color (n = 352)	
Blanco	181 (51,4)
Pardo/negro	163 (46,3)
Otros	8 (2,3)
Nivel de escolaridad (n = 281)	
Primaria	146 (52,0)
Secundaria	77 (27,4)
Superior	40 (14,2)
Postgrado	1 (0,4)
No alfabetizado	17 (6,0)
Comorbilidades (n = 356)	
Si	304 (85,4)
No	52 (14,6)

NEWS 2 y NEWS A mostraron una alta sensibilidad para identificar la IARP. Por otro lado, MEWS y qSOFA fueron específicos (Tabla 2).

**Tabla 2:** Sensibilidad y especificidad de las puntuaciones NEWS 2, NEWS A, MEWS, qSOFA para identificar insuficiencia respiratoria aguda, shock y parada cardiopulmonar – São Paulo, SP, Brasil, 2025.

	Insuficiencia respiratoria aguda				Shock				Paro cardiorrespiratorio			
	Si n (%)	No n (%)	S (%)	E (%)	Si n (%)	No n (%)	S (%)	E (%)	Si n (%)	No n (%)	S (%)	E (%)
NEWS 2												
Alto	172 (48,3)	116 (32,6)	88	28	7 (2)	281 (78,9)	88	19	2 (0,6)	286 (80,3)		
Bajo/medio	23 (6,5)	45 (12,6)			1 (0,3)	67 (18,8)			1 (0,3)	67 (18,8)	67	19
NEWS A												
Alto	180 (50,6)	133 (37,4)	92	17	8 (2,2)	305 (85,7)	100	12	2 (0,6)	311 (87,4)		
Bajo/medio	15 (4,2)	28 (7,9)			0 (0)	43 (12,1)			1 (0,3)	42 (11,8)	67	12
MEWS												
Alto	36 (10,1)	21 (5,9)	19	87	5 (1,4)	52 (14,6)	63	85	0 (0)	57 (16)	0	84
Bajo	159 (44,7)	140 (39,3)			3 (0,8)	296 (83,1)			3 (0,8)	296 (83,1)		
qSOFA												
Alto	32 (9)	21 (5,9)	16	87	5 (1,4)	48 (13,5)	62	86	1 (0,3)	52 (14,6)	33	85
Bajo	163 (45,8)	140 (39,3)			3 (0,8)	300 (84,3)			2 (0,6)	301 (84,6)		

Clave: MEWS: *Modified Early Warning Score*; qSOFA: *quick Sequential Organ Failure Assessment*; NEWS2: *National Early Warning Score versión 2*; NEWS A: *National Early Warning Score Age*; S: sensibilidad; E: especificidad.

En cuanto a la identificación del shock, NEWS 2 y NEWS A mostraron una alta sensibilidad. MEWS y qSOFA mostraron una buena especificidad (Tabla 2).

En cuanto a la PCR, NEWS 2 y NEWS A mostraron una sensibilidad media. Sin embargo, MEWS y qSOFA mostraron una buena especificidad (Tabla 2).

En cuanto a la identificación de la necesidad de ingreso en la UTI, NEWS 2 y NEWS A presentaron valores de sensibilidad más elevados. Por el contrario, MEWS y qSOFA tuvieron una sensibilidad más baja (Tabla 3).

**Tabla 3:** Sensibilidad y especificidad de las puntuaciones NEWS2, NEWS-A, MEWS, qSOFA para identificar el ingreso en la Unidad de Cuidados Intensivos y la muerte – São Paulo, SP, Brasil, 2025.

		Internación en UTI						Muerte				
		Si n (%)	No n (%)		S (%)	E (%)		Si n (%)	No n (%)		S (%)	E (%)
NEWS 2												
Alto		157 (44,1)	131 (36,8)		89	27		87 (24,4)	201 (56,5)		87	22
Bajo/medio		2 (5,6)	48 (13,5)					13 (3,7)	55 (15,4)			
NEWS A												
Alto		165 (46,3)	148 (41,6)		93	17		92 (25,8)	221 (62,1)		92	14
Bajo/medio		12 (3,4)	31 (8,7)					8 (2,2)	35 (9,8)			
MEWS												
Alto		37 (10,4)	20 (5,6)		21	89		20 (5,6)	37 (10,4)		20	86
Bajo		140 (39,3)	159 (44,7)					80 (22,5)	219 (61,5)			
qSOFA												
Alto		35 (9,8)	18 (5,1)		20	90		24 (6,7)	29 (8,1)		24	89
Bajo		142 (39,9)	161 (45,2)					76 (21,3)	227 (63,8)			

Clave: MEWS: *Modified Early Warning Score*; qSOFA: *quick Sequential Organ Failure Assessment*; NEWS 2: *National Early Warning Score versión 2*; NEWS A: *National Early Warning Score Age*; S: sensibilidad; E: especificidad; UCI: Unidad de Cuidados Intensivos.

NEWS 2 y NEWS A tienen una alta sensibilidad para identificar la muerte. Por otro lado, MEWS y qSOFA tienen una mayor especificidad (Tabla 3).

Se realizó un análisis complementario para evaluar la concordancia entre el deterioro clínico y qSOFA y MEWS, que dio como resultado un coeficiente Kappa de 0,269 (IC 95%: 0,21 a 0,4; valor p ≤ 0,001), lo que indica una concordancia deficiente. La concordancia entre el deterioro clínico y NEWS 2 y NEWS A dio lugar a un coeficiente Kappa de 0,677 (IC del 95%: 0,61 a 0,8; valor de p ≤ 0,001), considerado bueno.

## DISCUSIÓN

En este estudio se investigó la aplicación de los PAT en la atención de pacientes con COVID-19. Se evaluó la eficacia de estas puntuaciones en la identificación precoz de los deterioros clínicos y sus resultados en un entorno hospitalario. Este enfoque pone de relieve la importancia de los PAT como herramientas de seguimiento y gestión clínica eficaz durante la pandemia.

La población estaba compuesta mayoritariamente por hombres, con una edad media de 59,4 años, de raza blanca y con estudios primarios incompletos. Los estudios de perfil epidemiológico de los pacientes hospitalizados por COVID-19 apuntan a una edad media similar^(17)^, lo que sugiere que la edad es un factor de riesgo para el ingreso hospitalario, ya que la población de mayor edad muestra una disminución del sistema inmunitario, así como de las capacidades respiratorias, cardiovasculares y de respuesta inflamatoria.

La mayoría de los pacientes hospitalizados por COVID-19 presentaban dos o más comorbilidades, siendo la hipertensión arterial sistémica la más prevalente, seguida de la diabetes mellitus. La COVID-19 tiene una alta tasa de letalidad en individuos mayores de 60 años^(18)^, observándose que la presencia de una o más comorbilidades en esta población es más prevalente. En un informe sobre 355 muertes por COVID-19 en Italia, la edad media era de 79,5 años y aproximadamente la mitad de la muestra (48,5%) presentaba tres o más comorbilidades, y estos factores fueron decisivos en la tasa de letalidad^(19)^. En otro informe en el que se incluyeron casi 300.000 casos de COVID-19 en Estados Unidos, la tasa de hospitalización fue seis veces superior y la tasa de mortalidad 12 veces superior entre los que tenían alguna comorbilidad en comparación con los que no la tenían^(20)^.

Los pacientes con COVID-19 pueden evolucionar rápidamente a IRA grave, shock y PC. En este estudio, el 26,7% de los pacientes evolucionaron a insuficiencia respiratoria grave, el 32,6% a shock y el 27% a parada cardiaca. Así pues, la identificación precoz y el tratamiento oportuno de los casos son cruciales para un buen resultado, al igual que el uso de puntuaciones de deterioro clínico, como NEWS 2, al ingreso del paciente, que han demostrado tener un alto efecto predictivo de hospitalización y muerte^(21)^.

Los PAT, en general, pueden constituir Sistemas de Respuesta Rápida con el objetivo de sistematizar el reconocimiento precoz de los signos de deterioro clínico y desencadenar un manejo adecuado^(22)^. Aunque la mayoría de los estudios sobre la eficacia de los PAT en la reducción de la mortalidad carecen de una metodología coherente, su implantación puede reducir la mortalidad y los ingresos no planificados en UCI^(22)^. Sin embargo, la implantación de los PAT en las unidades de urgencias ha sido recomendada y ampliamente utilizada durante la pandemia de COVID-19^(23)^.

En nuestro estudio, NEWS 2 y NEWS A mostraron una buena sensibilidad, ya que identificaron a los pacientes con alto riesgo de deterioro clínico, ingreso en UCI y mortalidad, pero, por otro lado, presentaron un alto grado de falsos positivos. Un análisis retrospectivo publicado en el Reino Unido mostró que NEWS 2 ≥ 5 era muy sensible y poco específico para predecir el deterioro clínico^(24)^. En un estudio de cohortes destinado a evaluar el rendimiento de NEWS y NEWS 2 en relación con el desenlace de muerte y hospitalización inesperada en pacientes COVID-19, las puntuaciones analizadas obtuvieron buenos resultados^(25)^. En una cohorte multicéntrica se llegó a la conclusión de que la capacidad predictiva de NEWS 2 y sus variantes en relación con los acontecimientos graves y la muerte es cuestionable^(26)^ y, a pesar del aumento de la edad, NEWS A obtiene mejores resultados^(27)^.

En contraste con NEWS 2 y NEWS A, tanto qSOFA como MEWS mostraron una sensibilidad baja y una especificidad alta, aunque una cohorte retrospectiva que incluyó constantes vitales prehospitalarias mostró una sensibilidad alta de qSOFA para predecir el ingreso en la UCI y la mortalidad. Según los autores, qSOFA > 1 representaba una probabilidad de muerte 58,1 veces mayor^(28)^. Hay que tener en cuenta que ambas puntuaciones no incluyen la saturación de oxígeno como variable y que la insuficiencia respiratoria aguda fue una complicación frecuente del COVID-19, siendo sus variables más sensibles al shock hemodinámico, lo que puede justificar que en este estudio NEWS 2 y NEWS A mostraran mejor concordancia con el resultado de deterioro clínico que MEWS y qSOFA. Otro aspecto a tener en cuenta es que los EAP no se desarrollaron para predecir la mortalidad, sino el deterioro clínico.

Cuando comparamos el rendimiento de las puntuaciones en este escenario, nos parece claro que las puntuaciones que se basan en una combinación de frecuencia respiratoria y saturación de oxígeno aumentan la sensibilidad para detectar el deterioro clínico en situaciones en las que la IRpA es más frecuente. De hecho, en una publicación en la que se comparaban puntuaciones de deterioro se observó que NEWS 2 era la puntuación más sensible para predecir la forma grave del COVID-19 en comparación con otras puntuaciones aplicadas a pie de cama^(21)^. Un estudio de cohortes retrospectivo realizado en un hospital terciario de China, con 62.403 pacientes tratados en urgencias, demostró que NEWS 2 era significativamente mejor que MEWS y qSOFA para predecir la mortalidad en urgencias^(29)^. Por lo tanto, estos hallazgos sugieren que tanto NEWS 2 como NEWS A pueden ser una herramienta importante para optimizar el proceso de evaluación de la enfermera en la clasificación del riesgo y el seguimiento de los pacientes durante su estancia en el SE.

Nuestro estudio estuvo limitado por el hecho de que se llevó a cabo en un único centro y, en algunos casos, por el carácter incompleto de los registros de parámetros clínicos utilizados para calcular las puntuaciones de alerta. Sin embargo, se llevó a cabo en un hospital de referencia en la capital de São Paulo para la atención de casos sospechosos de COVID-19 durante la pandemia. Además, este estudio contribuye al avance de la enfermería en la mejora de la evaluación clínica, capaz de identificar rápidamente signos de deterioro clínico en pacientes con COVID-19; en la toma de decisiones basadas en la evidencia, para una atención más segura y de calidad; en la estandarización de los cuidados, garantizando que los profesionales utilicen criterios uniformes de monitorización e intervención; en la formación de los profesionales, aumentando el conocimiento y la capacidad del equipo para hacer frente a situaciones críticas; y en la reducción de la mortalidad y de las complicaciones a través del uso eficaz de las puntuaciones, beneficiando directamente al paciente y mejorando los indicadores de salud.

Creemos que, ante el riesgo de futuras pandemias, el tema debe seguir siendo relevante, ya que trata de cerca cómo se organizarán los profesionales de enfermería para dar una respuesta rápida y asertiva. En este contexto, creemos que es racional utilizar las mejores herramientas, fácilmente aplicables y de bajo coste, que mejoren el rendimiento de la detección del deterioro clínico y aumenten la seguridad en salud.

## CONCLUSIÓN

En este contexto, tanto NEWS 2 como NEWS A destacan entre los PATs y pueden ser útiles para subvencionar al personal de enfermería en la valoración del paciente, ya que tienen una alta sensibilidad, identificando de forma rápida y objetiva un posible deterioro clínico y permitiendo la aplicación precoz de medidas en la atención al paciente en un SEH. Por otro lado, MEWS y qSOFA deberían priorizarse para confirmar resultados negativos en no pacientes, ya que son más específicos a la hora de verificar el deterioro clínico en pacientes.

## Data Availability

El conjunto completo de datos que respaldan los resultados de este estudio se publicó en el propio artículo.
